# Investigating body patterning in aquarium-raised flamboyant cuttlefish (*Metasepia pfefferi*)

**DOI:** 10.7717/peerj.2035

**Published:** 2016-05-17

**Authors:** Amber Thomas, Christy MacDonald

**Affiliations:** The Seas, Epcot, Walt Disney World Resort, Disney’s Animals, Science and Environment, Lake Buena Vista, FL, United States

**Keywords:** Body patterning, Cuttlefish, *Metasepia pfefferi*, AutoClass, Communication, Behavior

## Abstract

Cuttlefish are known for their ability to quickly alter their total appearance, or body pattern, to camouflage or to communicate with predators, prey and conspecifics. The body patterns of some species have been extensively documented to gain a better understanding of their behaviors. However, the flamboyant cuttlefish (*Metasepia pfefferi*) is largely unstudied. Recently, aquarists have been able to breed, house and display *M. pfefferi*, giving researchers ample opportunities to study their behavior under those conditions. This study aimed to identify the dorsally-visible components of the body patterns used by 5 sexually-mature, freely-behaving, F5 generation *M. pfefferi* in their home aquarium at The Seas in Epcot at Walt Disney World Resorts^®^, Lake Buena Vista, FL, USA. Furthermore, we aimed to determine the most probable patterns used by this population of animals and to create a database of components that can be used in future behavioral studies. We found that this population of *M. pfefferi* use a combination of 7 textural, 14 postural, 7 locomotor and between 42 and 75 chromatic components in their home aquarium. Using maximum likelihood analysis and AutoClass@IJM software, we found that these components combine to generate 11 distinct body patterns. The software was able to sort 98% of the live animal observations into one of the 11 patterns with 90% confidence and 88% of observations with 99% confidence. Unusually for cuttlefish, 8 of the 11 identified patterns contained at least one “traveling” component (i.e., traveling waves or blinking spots) in which the colors on the skin appeared to travel on the animal’s mantle. In other species, these components are generally seen during hunting or aggression, but this population of *M. pfefferi* uses them frequently during a variety of contexts in their home aquarium. With few published data on the behavior of *M. pfefferi* in their natural environment, we cannot compare the behavior of the tank-raised individuals in this study to animals in the wild. However, this study provides the groundwork necessary for future studies of *M. pfefferi* body patterning and behavior.

## Introduction

Coleoid cephalopods (octopus, squid and cuttlefish) are a diverse group of animals that are well known for their ability to rapidly alter their appearance. To accomplish this rapid polyphenism, coleoids possess several anatomical structures that are functionally unique to this group, the most notable of which is the chromatophore. This organ in the skin consists of a compartment of pigment granules surrounded by neurally-controlled, radial muscle fibers. The expansion of these muscle fibers leads to the exposure of the pigment granules and the outward appearance of a particular hue on the animal’s skin (for review, see Messenger, 2001). The available hues of pigment granules vary slightly among species (*Loligo opalescens* and *Sepia officinalis*: yellow, red and brown (Cloney & Florey, 1968; Hanlon & Messenger, 1988); *Alloteuthis subulata*: yellow and red (Cornwell, Messenger & Hanlon, 1997); *Octopus vulgaris*: yellow, orange, red, brown and black Packard & Hochberg, 1977). The arrangement of chromatophores varies among different areas of the body but this variation is consistent among individuals of the same species (Hanlon & Messenger, 1988; Packard & Hochberg, 1977). When the chromatophores are retracted, layers of reflective iridophores and leucophores are exposed. These cells are responsible for the iridescent blues and greens (iridophores) and the high contrast whites (leucophores) observed on the animal’s skin (for review, see Messenger, 2001).

The individual chromatophores and reflecting cells as well as the photophores, muscles and internal organs that aid in the animal’s polyphenism are considered “elements.” They form the foundation of the hierarchy used by scientists to understand and describe cephalopod body patterning. In this hierarchy, the level that represents a grouping of elements is termed a “unit.” For chromatophores, units can be described in two ways: “morphological units” and “physiological units.” Morphological units describe an anatomical grouping of elements whereas physiological units describe the groupings of chromatophores that are simultaneously stimulated by the central nervous system (Packard, 1982). Groups of units are called “components” which were originally described as “the parts that go to make up the whole” (Packard & Sanders, 1971). Components are broken into four categories: chromatic, textural, postural or locomotor. When all components simultaneously expressed by an animal are combined and seen as a whole, it is termed a “body pattern” (Hanlon & Messenger, 1988; Packard & Hochberg, 1977; Packard & Sanders, 1971). This hierarchical order allows a description of a whole animal’s body pattern to reflect the macroscopic components as well as the microscopic elements and units.

The body patterns described above can be used to avoid detection, recognition or capture by predators or for communicatory signals between conspecifics, predators or prey (for review, see Hanlon & Messenger, 1996). Despite the vast number of potential uses, the patterns exhibited by any cephalopod species is considered “finite” or “fixed.” In general, ethologists report body pattern repertoires ranging from 2 to 16 patterns per species and each pattern may be classified as chronic (displayed for minutes to hours) or acute (displayed for seconds to minutes) (Table 3.2 (Hanlon & Messenger, 1996)). Body patterns are also species-specific, allowing taxonomists to differentiate between species that look similar (Hanlon, 1988).

Cephalopod body patterns have been extensively described for many species: Humboldt squid, *Dosidicus gigas* (Trueblood et al., 2015); Tropical Arrow Squid, *Doryteuthis plei* (Postuma & Gasalla, 2015); Larger Pacific Striped Octopus (Caldwell et al., 2015); Opalescent Inshore Squid, *Doryteuthis opalescens/Loligo opalescens* (Hunt et al., 2000); Longfin Inshore Squid, *Doryteuthis pealeii*/*Loligo pealeii* (Hanlon et al., 1999); Common Squid, *Loligo vulgaris reynaudii* (Hanlon, Smale & Sauer, 1994); Common Cuttlefish, *Sepia officinalis* (Hanlon & Messenger, 1988); and Common Octopus, *Octopus vulgaris* (Packard & Sanders, 1971) (reviewed by Borrelli, Gherardi & Fiorito, 2006; Hanlon & Messenger, 1996). These body patterns were described using extensive photographic and video recording, experiments in the field and in the laboratory, intensive cataloging of all of the components present at a given time, and the human’s ability to recognize recurring patterns. For the experienced ethologist, recognizing body patterns in this way presents few difficulties, but the subjective manner of these descriptions makes recognition for new observers and ethologists in other laboratories more difficult. Humans also tend to see patterns where none exist (a condition termed “apophenia”) and overlook patterns where large amounts of data are involved. Therefore, Crook, Baddeley & Osorio (2002) suggested an alternative method for body pattern classification in which an observer records the presence or absence of chromatic, postural, textural and locomotor components that are concurrently exhibited by a live cephalopod. The data collected from hundreds of these live animal observations are then analyzed using an automated signal classification system in which clusters (i.e., body patterns) are generated based on the statistical likelihood that (i) a combination of components are frequently expressed together; and (ii) each body pattern is expressed frequently enough to warrant its own cluster. This method was used to identify the number of body patterns frequently used in the common cuttlefish, *S. officinalis* (Crook, Baddeley & Osorio, 2002), but has not been used in other cephalopod species.

One potential candidate for this system of automated body pattern classification is the flamboyant cuttlefish, *Metasepia pfefferi*. Although *M. pfefferi* was initially discovered in 1885 (Hoyle, 1885), the first observations of living individuals were not reported until 1988 (Roper & Hochberg, 1988) and few studies have been published regarding this species since then. In the wild, *M. pfefferi* are found in the tropical, shallow (<85 m) waters around Australia, Papua New Guinea and Indonesia. These benthic cephalopods have been observed hunting fish and crustaceans on sandy or muddy substrates and laying their eggs in crevices of rock, coral, wood or coconut husks (Reid, Jereb & Roper, 2005; Roper & Hochberg, 1988). The two specimens observed by Roper & Hochberg were alone when captured (1988), but, to our knowledge, no further published documentation of their natural social behaviors or population densities exist. Roper & Hochberg (1988) also provide the only published report of *M. pfefferi* body patterning, describing the “extremely rich repertoire of components” observed in this species. However, they note that their observations were “brief and hence represent only a preliminary inventory of the chromatic, textural and postural components shown by this species”. Studies on the only other species in the same genus, *Metasepia tullbergi*, are also limited, but initial descriptions of both species indicate that their morphology, behavior, mode of locomotion and their “striking and colorful” body patterning distinguish these species from the well-studied cuttlefish in the genus *Sepia* (Adam & Rees, 1966; Roper & Hochberg, 1988). Despite the lack of data regarding wild *M. pfefferi*, aquarists have begun to breed and display this species in public aquariums (Grasse, 2014). This study aims to (1) expand upon the preliminary database of chromatic, textural, postural and locomotor components used by freely behaving, tank-raised *M. pfefferi* in their home aquarium; (2) develop an interactive and iterative database of the components identified which can easily be amended for future studies; (3) utilize an automated signal classification system to determine the number of commonly used patterns in tank-raised *M. pfefferi*, and the components that make up those patterns; (4) provide the groundwork necessary for future studies of *M. pfefferi* body patterning.

## Methods

### Animal husbandry

Five tank-raised (F5 generation), sexually-mature *M. pfefferi* were kept in a public display aquarium at The Seas in Epcot at Walt Disney World Resorts^®^, Lake Buena Vista, FL, USA. This population contained three females (approximate mass: 20 g) and two males (approximate mass: 10 g) 3–4 months of age. Their aquarium enclosure included a cylindrical acrylic tank (1.2 m diameter, 0.6 m high, and 550 L total system volume) with a closed-loop filtration system. This filtration system contained an undergravel filter with a pleated canister filter for mechanical filtration, activated carbon and protein skimming for chemical filtration and a trickle filter for biological filtration. The animals were provided a low contrast, off-white or light gray, medium-sized (approximately 1 cm × 1 cm) crushed coral substrate. Habitat including artificial resin coral décor, plastic coconut huts (All Living Things^®^ Coconut Shell Reptile Ornament; Petsmart Inc., Phoenix, AZ, USA) and *Tridacna* clam shells were offered to provide egg laying surfaces and areas of rest. This tank was illuminated using 2 compact fluorescent lamps with dual actinic light (peak wavelength: 460 nm, measured intensity: 1,100–2,400 lux) with a 14:10 h on:off light cycle to accommodate public viewing. The feeding regimen consisted of offering live *Paleomonetes* sp. shrimp 3 times per day and 2–4 shrimp per animal per feed. Water quality and tank cleanliness were maintained through regular water changes, gravel washes, and habitat cleaning. Water quality parameters were monitored and maintained for the health of the animals. Water temperature of this system was 24–26 °C, salinity was 33–36 ppt, and pH was 8.00–8.20. Nitrogenous compounds including ammonia, nitrite and nitrate were maintained at NH_3_ < 0.01 mg L^−1^, NO_2_ < 0.10 mg L^−1^, and NO3 < 25 mg L^−1^ due to the specific sensitivity of this species.

### Video collection

To avoid moving cuttlefish, all five animals were kept together, in the same physical and social environment that they were accustomed to and permitted to behave freely within the confines of the aquarium. All recordings were made via a small GoPro Hero 3™ Black camera (GoPro, Inc., San Mateo, California, USA) manually held just below the surface of the water. This set-up, while not ideal for this type of behavioral observation, was similar to routine events for the animals and therefore expected to have minimal effects on behavior. More specifically, these animals have been on public display since hatching and exposed daily to people (both guests and animal-care staff) and to cameras both above and beside their home aquarium. The animal-care staff reached their arms into the environment to clean, move refugia and remove eggs as needed, so the presence of a human hand or arm in their environment was not unusual. Also, holding the camera allowed for quick removal of the equipment if the animals showed signs of disturbance (e.g., inking, jetting into walls, alarm towards the camera or fast retreat into refugia). The recordings captured behaviors normally exhibited by the animals in this aquarium environment, including locomotion, feeding, mating and conspecific aggression. Although aspects of their home aquarium environment (e.g., lighting, substrate, shelter objects, conspecific density and disturbance frequency) are not typical of the natural habitat of *M. pfefferi*, this study was designed to record the components and patterns of healthy, reproducing individuals that were habituated to these particular conditions during their complete lifetime.

The camera was set to record at 1080 resolution and 48 frames second^−1^ and placed in a waterproof housing. Recordings were made opportunistically between 8:00 and 15:00 over a two-week period in July 2014. Guests were not present in the area until after 9:00 AM. Each recording session lasted between 5–10 min and in total, 71 min of video were collected. The recorded videos were reviewed and 1-s clips were extracted using Adobe Premiere Pro^®^ (v. CS6; Adobe, San Jose, California, USA) at a down-sampled rate of 30 frames second^−1^. Component analysis (as described below) was conducted on clips that met the following criteria: at least one individual was completely visible during the entirety of the clip and the clip was not recorded within 5-s of another clip unless a new individual entered the video frame or the individual in the frame changed body patterns during that time.

### Identifying components

To determine which components *M. pfefferi* utilized in producing body patterns in their aquarium environment, one observer (AT) examined individual frames of the 1-s clips and sketched and described every unique component (defined below) to generate a list of overall components. This process continued until 50 randomly selected 1-s clips could be inspected with no observations of new components. The observer remained vigilant in looking for any missed components in the subsequent analyzed videos, but none were observed.

The following definitions were used when describing and identifying components:

**Textural component:** the “smoothness or papillation of the skin” (Hanlon & Messenger, 1996).

**Postural component**: the overall positioning of the arms, head and mantle in relation to each other.

**Locomotor component**: the method of movement or rest of the entire animal in physical space.

**Chromatic component:** any grouping of dark, pink, yellow or white coloration that occurred consistently and independently in the same relative position on multiple animals over multiple (≥5) observations.

In an effort to provide future researchers with data that can be recombined and reanalyzed to reflect new discoveries and definitions as well as compare to past and future studies, we report chromatic components in terms of overall color as seen by the human eye. Thus, chromatic components have been divided into four sub-categories: dark (brown, black or dark purple), white, pink and yellow. We do not differentiate between various levels of color gradation (e.g., light yellow components and dark yellow components are both “yellow”). Because no data exist regarding *M. pfefferi* morphological or physiological unit arrangement, we describe chromatic components in terms of their overall appearance on the live animal.

Our method of separating chromatic components into four categories as described above differs from the traditional method of separating chromatic components into two categories: light and dark. We separated components in this way because the definition of what constitutes a “light” or “dark” component vary among studies. Many studies define a “dark” component as one in which the chromatophores (of any pigment) are expanded or in which dark internal body parts are visible through the skin. Light components are described as those resulting from the full retraction of chromatophores and subsequent exposure of reflecting cells (Burford, Robison & Sherlock, 2014; Bush, Robison & Caldwell, 2009; Hanlon & Messenger, 1996; Hanlon, Smale & Sauer, 1994; Jantzen & Havenhand, 2003; Messenger, 2001). Alternatively, in an extensive study of * S. officinalis*, yellow chromatophore expansion was grouped with “light” components, rather than “dark” ones (Hanlon & Messenger, 1988). In a study of *Loligo pealeii*, “iridescent” components were grouped separately from other “light” components (Hanlon et al., 1999). Recently, “red” components were separated from other “dark” components when describing the repertoire of the Humboldt squid (*Dosidicus gigas*) (Trueblood et al., 2015) because those components were “bright crimson red, not dark or brown associated with dark.” In other ethograms, there does not appear to be a definition of how “light” and “dark” components were divided (Caldwell et al., 2015; Hunt et al., 2000; Roper & Hochberg, 1988).

To allow for comparisons between our data and those from previous studies, we present two lists of chromatic components: the “Expanded” list (components as defined above) and the “Condensed” list (defined below). To generate the Condensed list, we retrospectively combined components into light (yellow, white and pink) and dark categories, and consolidated components that have been presented as variations of a single component in previous studies.

To follow convention, we only capitalize the first letter of the first word of a component name. Similarly, “stripes” refer to longitudinal lines across the body, whereas “bands” are oriented transversely on the body or arms. Any discussions of “motion” or “movement” refer to the locomotor components of a pattern whereas “travel” refers to chromatic components that appear to change location during their expression.

### Creating an interactive, iterative database of components

**Figure 1:**
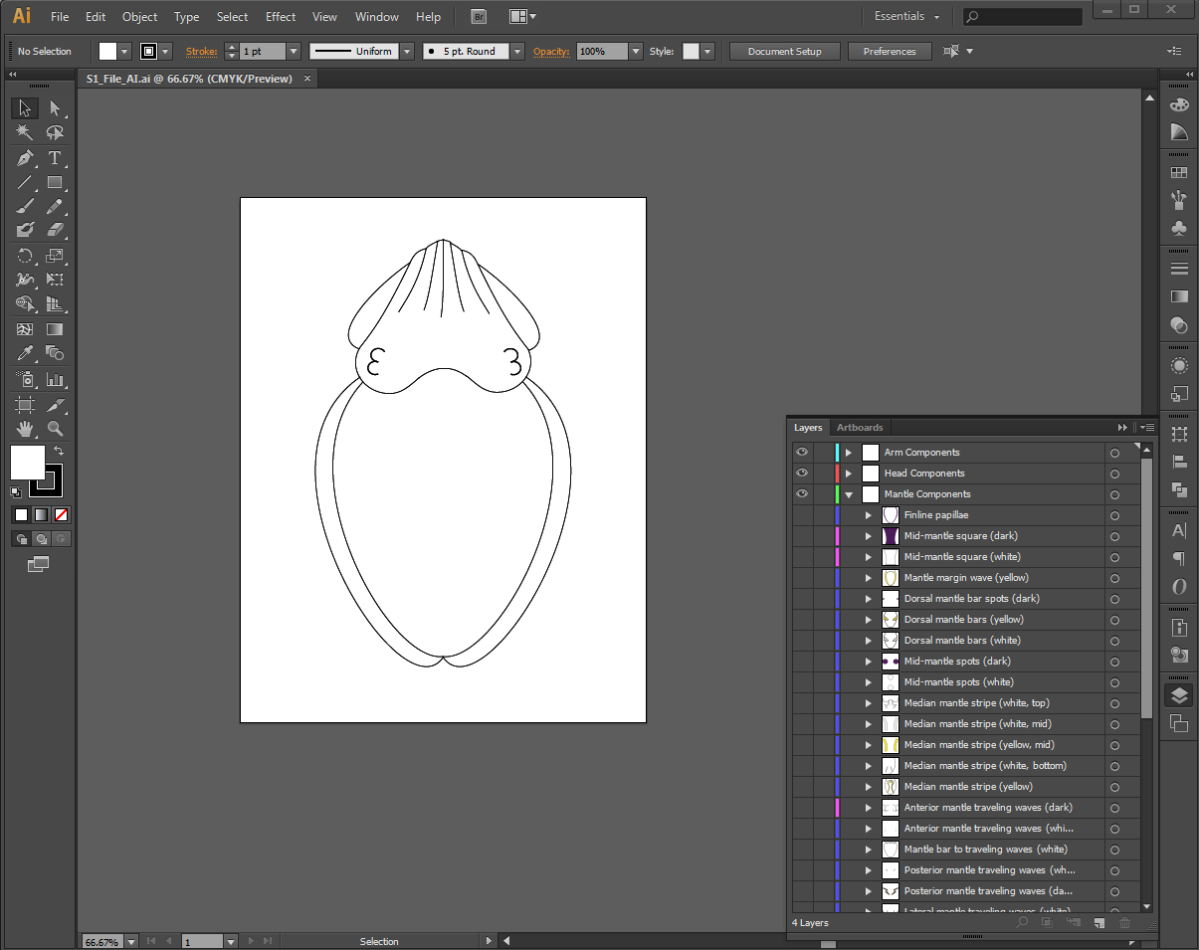
Screenshot of the interactive database for *Metasepia pfefferi* body patterning components. A screenshot of the interactive database created in Adobe Illustrator^®^ CS6 for *Metasepia pfefferi* body patterning components. On the right, the ‘layers’ panel is visible with Arm, Head and Mantle components which can each be made visible to recreate any observed body pattern. This database is available both as an Adobe PDF (File S1) and an editable Adobe Illustrator file (10.6084/m9.figshare.1509930).

Once the list and sketches of all components was complete, the components were each digitally illustrated utilizing Adobe Illustrator^®^ (v. CS6; Adobe, San Jose, California, USA) (Fig. 1). Similar to Adobe Photoshop^®^ (Adobe, San Jose, California), which was used to create a similar database for the Caribbean reef squid (*Sepioteuthis sepioidea*) (Byrne et al., 2003), Adobe Illustrator^®^ incorporates the function of ‘layers,’ allowing for the overall body pattern to be replicated by making some components visible and others invisible. It should be noted that the layers demonstrated in this database do not correspond to the levels of chromatophores or any cellular structure within the skin of *M. pfefferi*. For ease of use and future updates, all components are displayed on a cuttlefish with the same “Floating arm” posture. This position allows for the greatest visibility of all dorsal body parts to make the depiction of each component as clear as possible (Fig. 1). Any traveling components are illustrated with arrows to indicate their start and end points and their direction of travel. This database is available for download and use as a PDF (File S1) and an editable Adobe Illustrator^®^ File (10.6084/m9.figshare.1509930).

### Determining the number of body patterns

The 15th frame in each 1-s clip (see ‘Video Collection’) was evaluated to determine the presence and absence of each of the components on a binary basis as described by Crook, Baddeley & Osorio (2002) . If any part of the cuttlefish was obscured, the video clip was not used. The remaining frames in each 1-s clip were used to determine the presence of traveling components and the locomotor component. If multiple cuttlefish were in the frame of the video, they were scored individually, but any interaction (i.e., aggression or mating) between conspecifics was noted. Intra-observer variability was minimized by conducting all clip analysis within a 3-mo time span.

**Figure 2:**
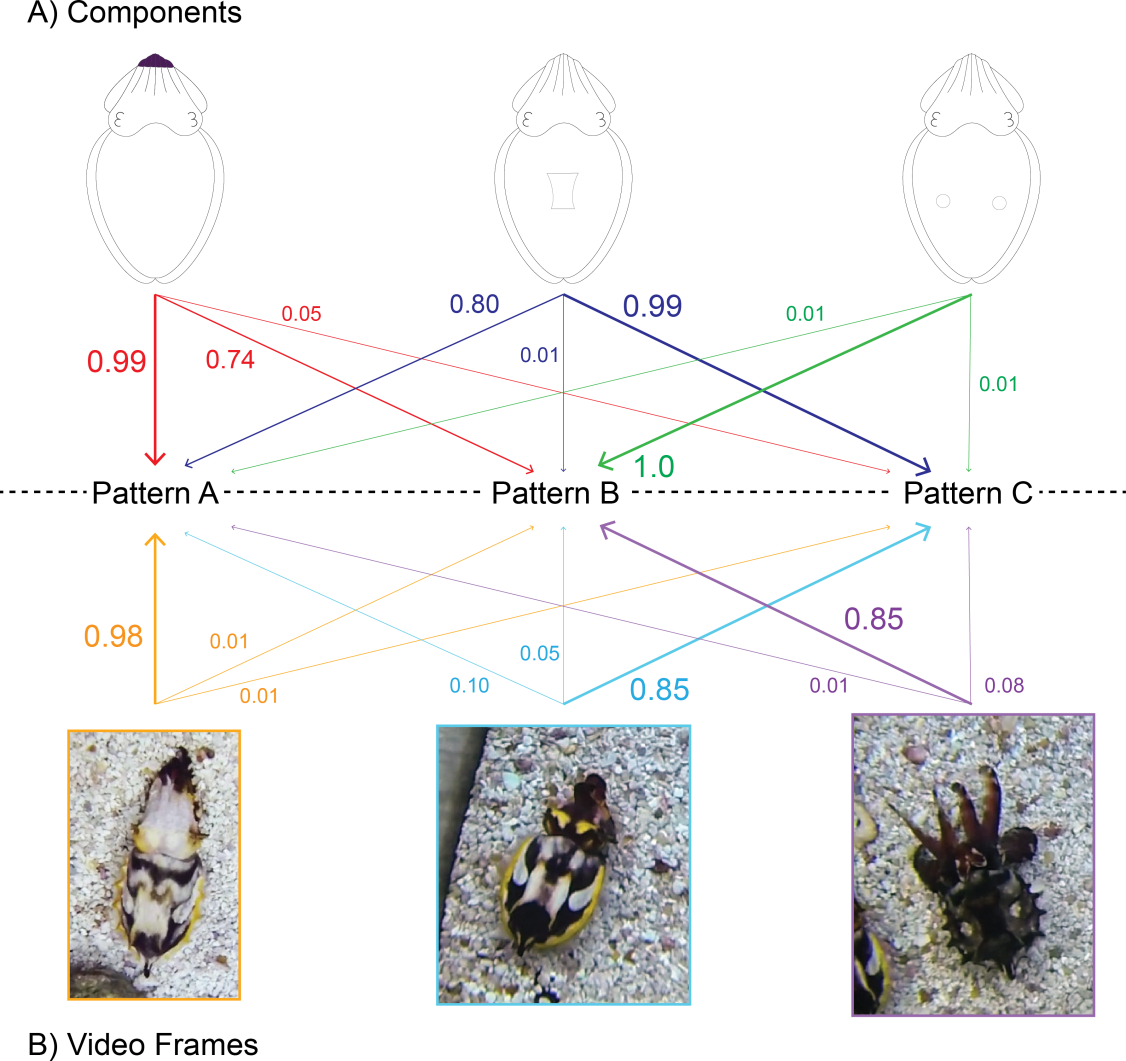
Diagrammatic explanation of the automated signal classification system. The probabilities that a given component will appear in a pattern (A) and that a specific 1-s clip can be grouped within that pattern (B). Arrow direction represents the grouping of a single component or 1-s clip into one of the three patterns. In this representation, arrow thickness denotes the probability that a component or 1-s clip is found within that pattern. Note that the probabilities expressed in (A) are independent of one another, for example, the probability of dark Arm tips appearing in animals grouped in Pattern A does not impact the probability of dark Arm tips appearing in Pattern B. On the other hand, the probabilities expressed in (B) are related and sum to 1, for example, the first 1-s clip image has a 98% chance that it belongs in the group “Pattern A,” and only a 1% chance that it should be grouped within Patterns B or C. The patterns, components and probabilities expressed in this figure are diagrammatic and do not indicate actual values.

This dataset was analyzed using the AutoClass@IJM web server (available at: http://37.60.156.20//autoclass/AutoclassAtIMRB.html) which utilizes maximum likelihood analysis to determine the most probable number of patterns and the most probable components present in each pattern (Achcar, Camadro & Mestivier, 2009) (Fig. 2A). This methodology prevents “overfitting” of data by assigning each individual 1-s clip a probability that it belongs within a given pattern (Fig. 2B). The web server of this program automatically chooses the most probable of 100 classifications on each run of the program (Achcar, Camadro & Mestivier, 2009; Crook, Baddeley & Osorio, 2002). Due to its probabilistic nature, the program was run with the dataset 100 times (for a total of 10,000 independent classifications) to determine the most probable number of patterns present. Once the number of patterns was determined, the “likeliest” set of patterns was chosen from within those results. The “likeliest” set of patterns was that which contained the highest number of 1-s clips that could be placed within a single pattern with a >90% probability that it belonged within one pattern as opposed to another.

**Figure 3:**
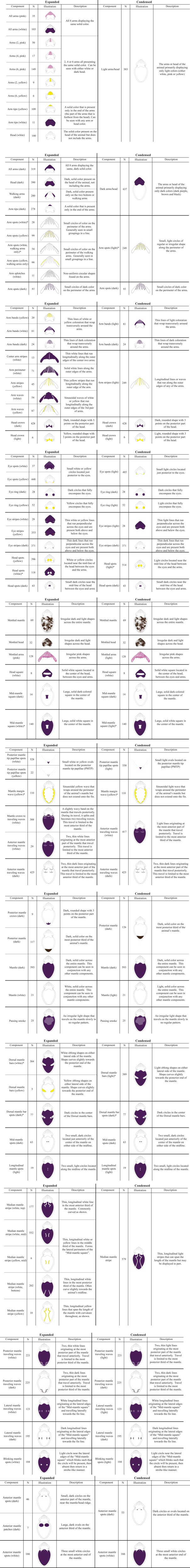
Comparison of the expanded and condensed lists of chromatic components. In this study, we provide both an Expanded List and a Condensed List of chromatic components for *M. pfefferi* (see ‘Methods’ for details). In the “Expanded” half of this figure, “Component” refers only to chromatic components as defined in the Methods: “Identifying Components” section. In the “Condensed” half of this figure, components from the Expanded List have been combined to reflect previously published ethograms of other species. *N* indicates the number of 1-s video clips that contained an animal displaying the specific component. All illustrations are taken directly from our Interactive Database and represent each component. For components on the arms or head, the mantle is not included in the illustration. White components are displayed on a dark background for visibility. Any arrows on the illustration indicate the direction and start and end locations for traveling components. The components on the Condensed List are displayed next to all of the Expanded components that were combined for its definition. For example, Dark arms/head on the Condensed List contains all of the All arms (dark), Head (dark), Walking arms (dark) and Arm tips (dark) components from the Expanded List. ∗, also identified by Roper & Hochberg (1988).

Once the “likeliest” result was chosen, the probability that a particular component would be present in each of the patterns was calculated as i/t where i = the total number of times a component was present within a particular pattern and t = the total number of individual 1-s clips within that pattern. These probabilities could then be used to determine which components were most likely to be present in any given pattern. Any components that had a >50% probability of being present within a given pattern were determined to be present in that pattern, whereas any components that had 0–50% probability of being present were treated as individual differences and not parts of the given pattern.

### Ethics statement

This study was submitted for formal review through Disney’s Animal Care and Welfare Committee and was approved (#IR1505). This research was conducted with strict guidelines to immediately remove the cameras if the animals appeared to be disturbed in any way (e.g., inking, jetting, or approaching the camera) to ensure optimal welfare during this study. However, none of those behaviors were observed during data collection. Animals appeared to ignore the observer and the camera and behave normally (e.g., feeding, mating, swimming, walking, and laying eggs). The animals observed in this study continued to exhibit healthy behaviors (e.g., reproduction, live prey capture and consumption, and limited levels of conspecific aggression) throughout the course of this study and their entire life cycle. Each animal eventually died a natural, senescent death typical of the species in managed care.

**Figure 4:**
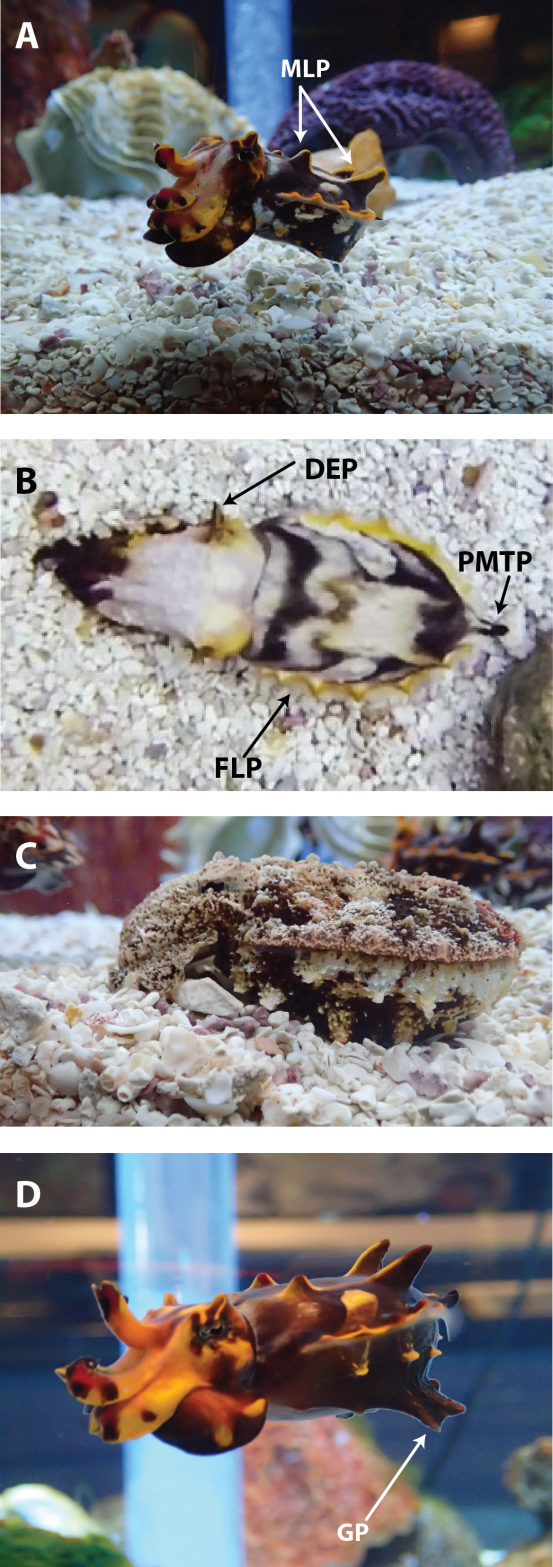
Skin textures expressed by *Metasepia pfefferi*. All seven skin textures exhibited by adult *M. pfefferi* in their home aquarium. These lateral images are used for descriptive purposes only and were not used for the dorsal-view observations reported in this study. (A) Major lateral papillae (MLP). Four MLP expressed in this photo, but the suppression of the anterior 2 MLP is also expressed. (B) Dorsal eye papillae (DEP), Posterior mantle tip papillae (PMTP), Finline papillae (FLP). (C) Coarse skin. (D) Glutapods (GP) though this animal is swimming, animals will use their glutapods to “walk” on the bottom of their environment, giving them the appearance of a tetrapod.

**Table 1:** Textural, postural and locomotor components expressed by *Metasepia pfefferi*. All postural, textural and locomotor components observed from this population of *Metasepia pfefferi* during the study period are listed and described. *N* refers to the number of 1-s video clips that contained an animal exhibiting a specific component.

	Component name	*N*	Description
Textural components	Posterior mantle tip papillae (PMTP)	639	Papillae extending laterally from the most posterior point of the mantle.
	Course skin	260	The skin appears to be rough or have small bumps on the surface.
	Dorsal eye papillae (DEP)	23^a^	Papillae extending transversely from the eyes.
	Glutapods^c^ (GP)		Extensions of the musculature and skin on the posterior, ventral end of the mantle and can be used to help aid in “walking”. Also called “Ambulatory flaps” (Roper & Hochberg, 1988).
	Major lateral papillae (2) (MLP)	239^a^	2 papillae extending perpendicular to the dorsal surface of the mantle, generally at the posterior corners of the Mid-mantle square.
	Major lateral papillae (4) (MLP)	280^a^	4 papillae extending perpendicular to the dorsal surface of the mantle at the corners of the Mid-mantle square.
	Major ventral papillae^c^		Papillae extending perpendicular to the ventral surface of the mantle but are not used for “walking”.
	Finline papillae (FLP)	329^a^	Papillae that extend around the perimeter of the mantle in a regular pattern and in the same plane as the fin.
	Smooth skin	288	No small bumps on the head or mantle.
	Ventral papillate skin^c^		The skin on the ventral surface of the mantle appears to have small bumps on the surface.
Postural components	“Elephant ear”	37	The walking arms (Arms IV) are set very wide and the other 6 arms extend further forward than Arms IV. Arms III extend the furthest.
	2 Raised arms	197^a^	The center 2 arms are raised higher than the remaining arms.
	4 Raised arms	82	The center 4 arms are raised higher than the remaining arms.
	All arms up and out (Circle)	15	All arms spread transversely in a circle such that each arm is perpendicular to the tank bottom.
	Concentric arms	33	All arms are held in line with the body and do not overlap but curve in towards the center, giving the appearance of several concentric circles.
	Drooping arms	145	All arms held perpendicular to the tank bottom.
	Flattened body	14^a^	Body is on the tank bottom and spread out so as to appear flat. Arms can be wide-splayed, concentric or “elephant ear” during this posture.
	Hovering arms	103	All 8 arms held in the same plane as the body.
	Raised head	2^a^	Animal sitting on the tank bottom and the head is lifted above the mantle.
	Spike arms	15	Six of the arms are extended to a point while the walking arms (Arms IV) are kept wide. Often a feeding posture where feeding tentacles extend beyond the tip of the six arms.
	Split arms	22	All arms are in line with the body but 4 arms are distinctly held to the left side of the animal and the other 4 are held to the right side.
	Elongate	92	All eight arms in front of head to make a narrow point.
	Wide bottom arms	16^a^	Walking arms are flattened out beneath the rest of the arms.
	Wide-splayed arms	50	All arms spread in front of the head in a haphazard fashion.
Locomotor components	Hovering	72^a^	Animal not resting on the tank bottom or other items, but instead is suspended above the tank bottom and remains motionless.
	Jetting	4^a^	The animal moves very quickly backwards but no ink is expelled.
	Sitting	188^a^	The animal is resting on the tank bottom or another item and remains motionless.
	Swimming	186^a^	Animal moves throughout the environment without touching the tank bottom or other items in the environment.
	Walking with tall arms	73^b^	Walking arms are extended perpendicular to the tank bottom, making the animal appear “tall”. The animal “walks” forward on these arms in a slow fashion.
	Walking with glutapods^c^		The animal uses the “glutapods” (see above) simultaneously with the walking arms to walk in a tetrapod-like manner.
	Walking with wide arms	132^b^	Walking arms curve slightly under the body and towards one another producing a wide surface on which to walk. The points of the two arms face each other but are present in two parallel planes such that one arm crosses over the other to “step” but the arms don’t touch.

**Notes.**

a described by Roper & Hochberg (1988).

b described by Roper & Hochberg (1988) as a single component instead of two separate components.

c Ventral components, observed but not included in the current study.

## Results

### Identifying components

#### Expanded list

**Figure 5:**
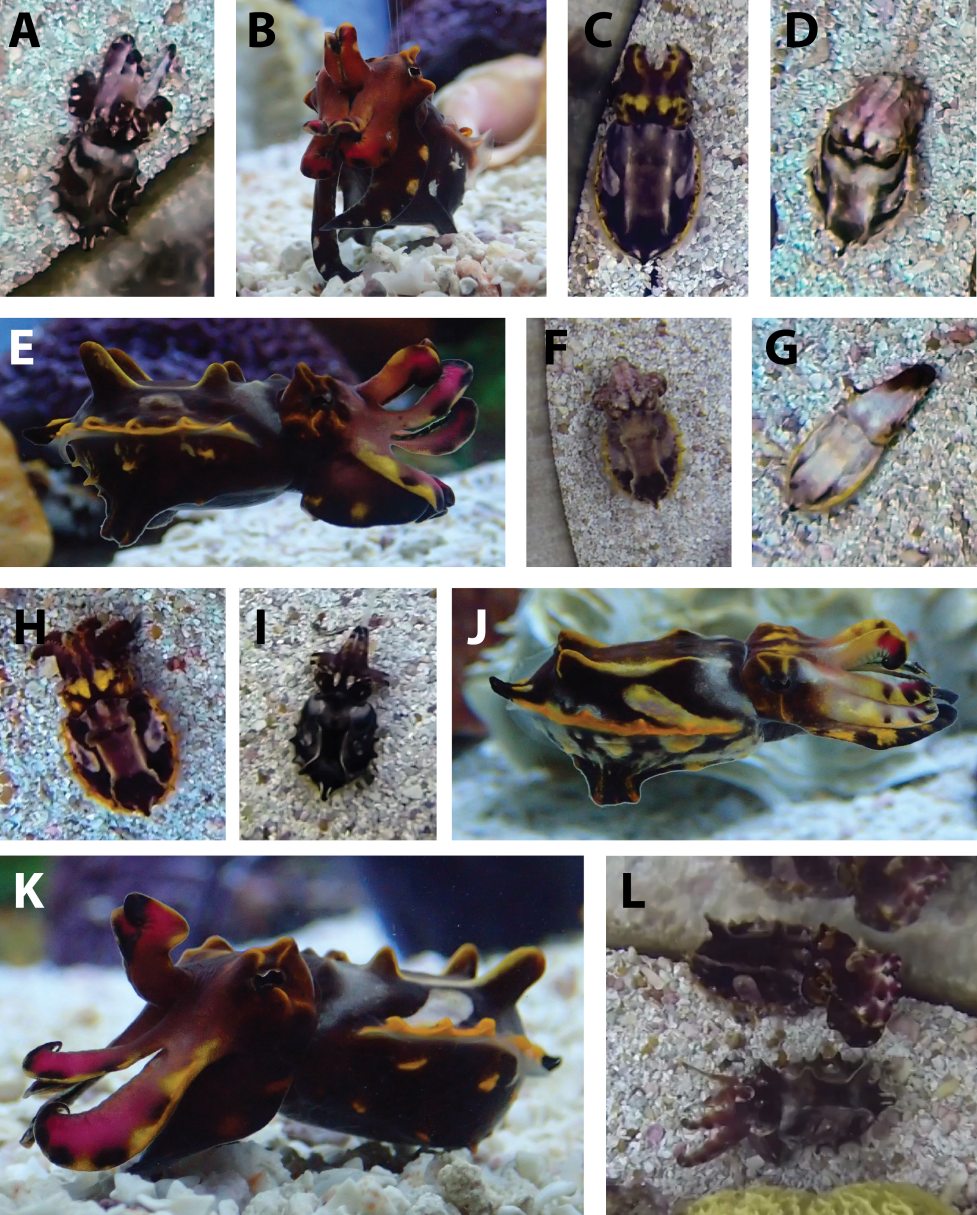
Postures expressed by *Metasepia pfefferi*. All 14 body postures identified in this population of *M. pfefferi* in their home aquarium. Dorsal-view images are screenshots from 1-s clips used for analysis in this study. Lateral-view images are for descriptive purposes only and were not included in our formal analysis. (A) Elephant-ear and Flattened body; (B) Raised head; (C) Split arms; (D) Drooping arms; (E) Four arms raised; (F) Wide bottom arms; (G) Elongate; (H) All arms up and out (Circle); (I) Spike arms (J) Hovering arms (K) Two arms raised (L) Concentric arms (top) Wide-splayed arms (bottom).

A total of 656, 1-s clips were analyzed and 75 chromatic (Fig. 3), 7 textural (Table 1, Fig. 4), 14 postural (Table 1 and Fig. 5), and 7 locomotor (Table 1, Videos S1 and S2) dorsally-visible components were observed, described and catalogued. For chromatic components, this number included 27 components on the arms, 16 components on the head and 32 components on the mantle. We also observed 4 distinct regions of “travel” in traveling waves on the mantle. For *M. pfefferi* a pair of wave sets (each containing 2–3 waves) began at the most anterior point of the mantle and traveled posteriorly to the White square in the center of the mantle. A second pair of wave sets began at the most posterior point of the mantle and traveled anteriorly to the White square. A third and fourth set began on the right and left sides of the White square and travel laterally towards the finline. In *M. tullbergi* a fifth set of waves was described as moving within the White square (Laan et al., 2014), but that was not observed in *M. pfefferi*. Full descriptions and frequency of observation for chromatic components, including all traveling components, is listed in Fig. 3. Observation frequency is not meant to indicate any quantification, but to provide an impression of the prevalence of some components.

As initially described by Roper & Hochberg (1988), *M. pfefferi* moves throughout its environment in a unique fashion: by “walking” or “ambling” along the bottom. We describe two variations of this walking behavior which differ in how the walking arms (Arms IV) are oriented in relation to the rest of the body during locomotion (Table 1, Videos S1 and S2). Other locomotor components observed in this population were the same as those described by Roper & Hochberg (1988).

Four of the postural components that we identified in this population of *M. pfefferi* were similar to those included in the initial species description (Two raised arms, Flattened body, Raised head and Wide bottom arms) (Roper & Hochberg, 1988). We also describe an additional 10 postural components exhibited by *M. pfefferi* in their home aquarium (Fig. 5 and Table 1). Similarly, the textural components Dorsal eye papillae, Major lateral papillae (2), Major lateral papillae (4) and Finline papillae were also described by Roper & Hochberg (1988). We described three additional, dorsally-visible textural components and three ventral textural components (Fig. 4 and Table 1) of which the latter were described but not included in our body pattern analysis.

#### Condensed list

By combining chromatic components to reflect the most conservative past practices, the total number of chromatic components produced by *M. pfefferi* is 42 (See Fig. 3 for all consolidations). To reflect an ethogram of *Loligo pealeii* (Hanlon et al., 1999), any light (pink, white or yellow) solid coloration on the arms or head (including all light variations of All arms, Arms (2, 4 and 6), Arm Tips and Head) were combined to a single component: Light arms/head. Similarly, all solid, dark coloration on the arms or head (including All arms, Head, Walking arms and Arm tips) was combined into “Dark arms/head”. All light spots and splotches on the arms were combined to “Arm spots (light)” in the same way that they were combined in an ethogram of *Doryteuthis plei* (Postuma & Gasalla, 2015). Just as bands with variations were combined into a single component in *Loligo pealeii* (Hanlon et al., 1999), any light stripes or waves along any of the arms were consolidated into “Arm stripes (light).” White and yellow variations of Eye spots, Eye stripes, Head spots, Arm bands, Posterior mantle tip papillae spots, and Dorsal mantle bars were each consolidated into “light” categories of the same name.

### Determining the number of body patterns

**Figure 6:**
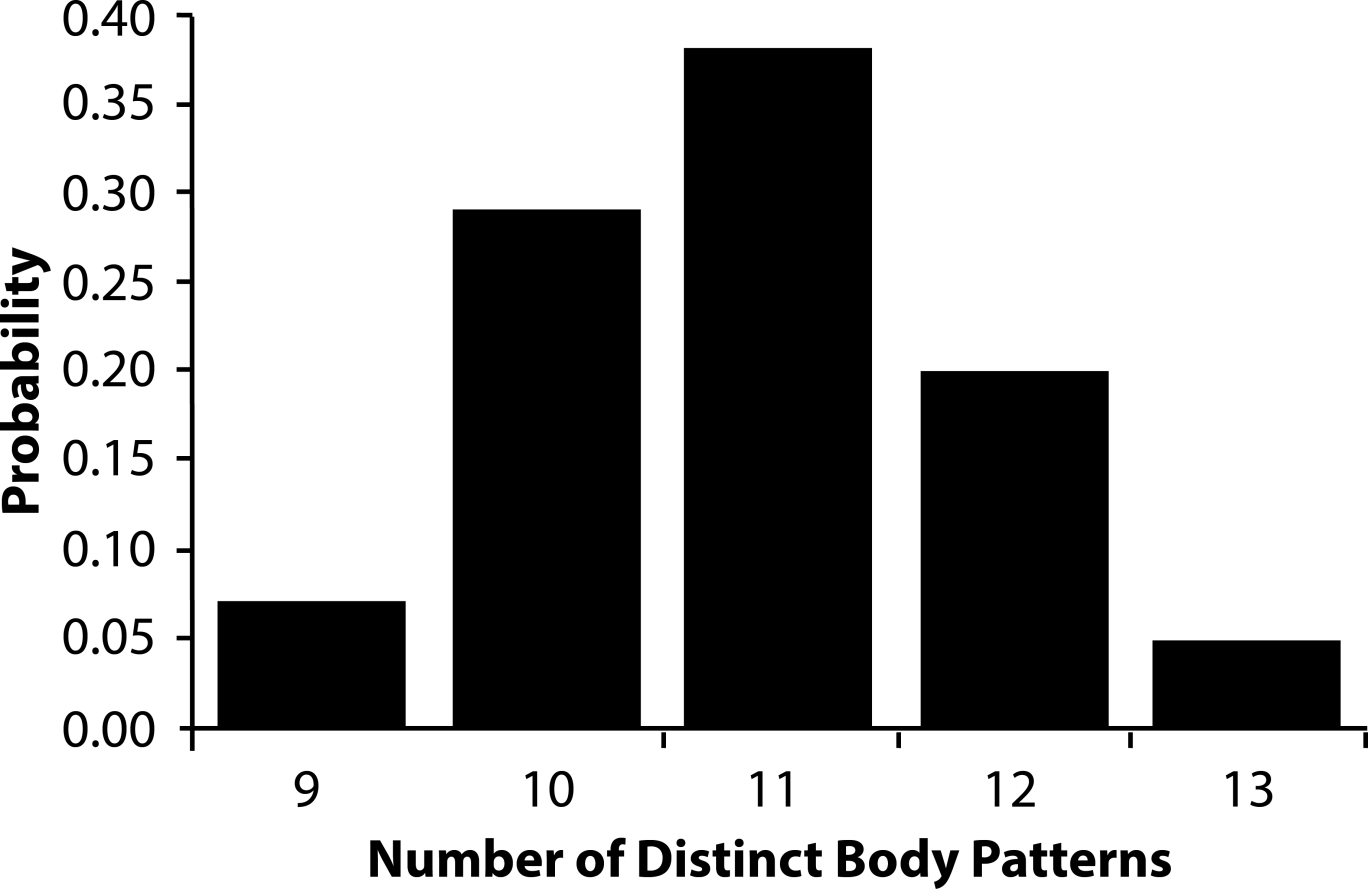
Relative probability of number of body patterns produced by *Metasepia pfefferi*. The relative probability that the number of distinct body patterns produced by *Metasepia pfefferi* is 9, 10, 11, 12, or 13. In 10,000 sorting attempts through the AutoClass@IJM software, there were no lower or higher estimated number of patterns.

The AutoClass@IJM webserver classified the 1-s clips into a range of 9–13 patterns (Fig. 6) where 11 patterns was the most frequent result. Within the results that contained 11 patterns, the most inclusive set allowed 98% of the clips to be sorted into one of the 11 patterns with >90% probability, and 88% of the clips to be sorted with 99% probability (Fig. 6).

**Figure 7:**
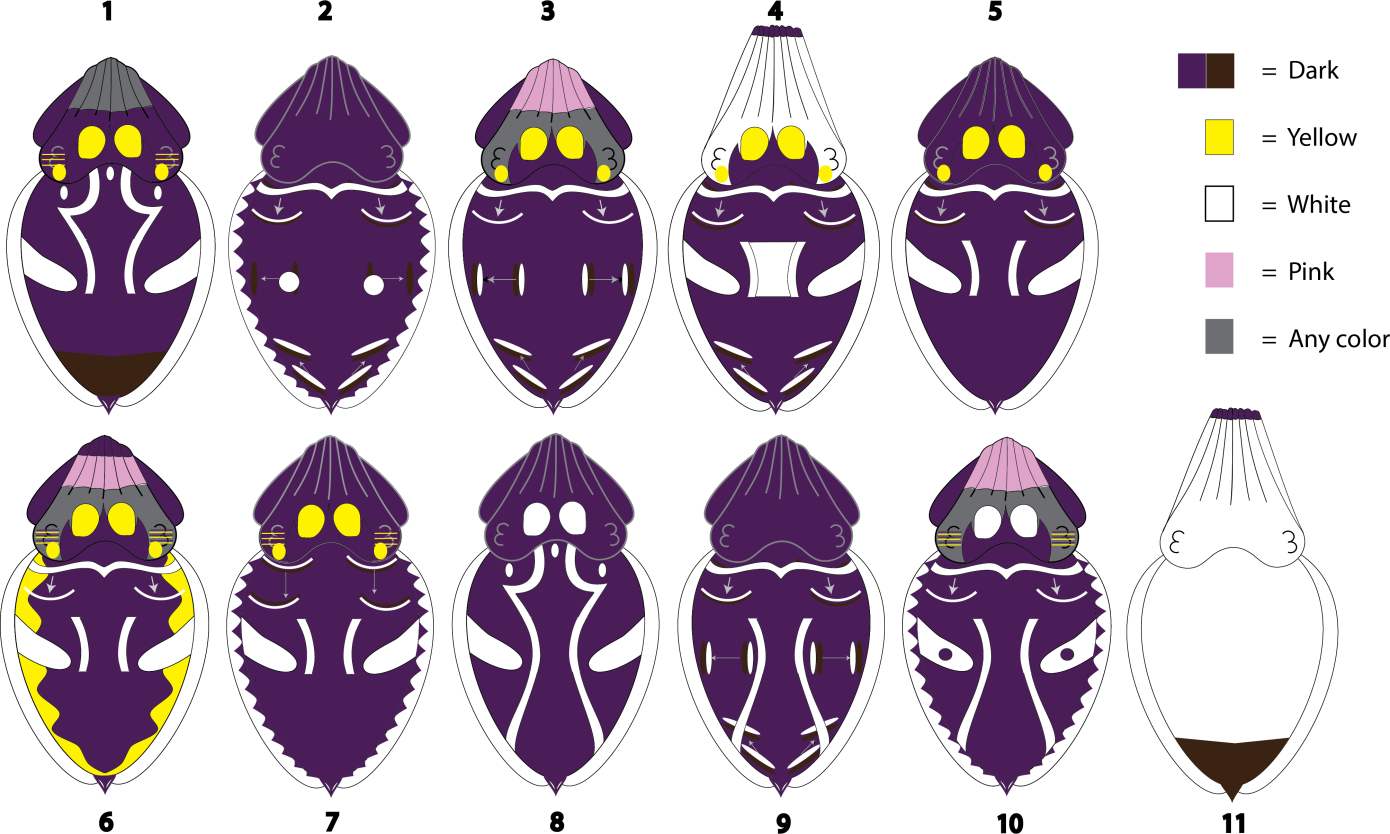
Body patterns produced by *Metasepia pfefferi*. The 11 patterns produced by freely behaving, sexually mature *Metasepia pfefferi* in their home aquarium as recognized by the AutoClass@IJM automated signal classification system. Each component included in a pattern depiction was observed in >50% of the 1-s clips sorted into that pattern category. As described in the text, each pattern is sorted into 4 color categories (as seen by the human eye): dark, yellow, white and pink. If no single color was observed in >50% of the 1-s clips for a certain body area (e.g., arms in Pattern 1 and head in Pattern 3) it is depicted as gray. Arrows on the illustrations indicate the direction and the start and end points for traveling components.

Of the 11 patterns identified (Fig. 7), eight contained at least one “traveling” component and three contained three “traveling” components. Only two patterns contained a postural component (Patterns 4 and 11 were both “Elongate posture”). Because the remaining patterns could be exhibited in a variety of postures, posture was considered individual variation for those specific body patterns. Four of the patterns contained a locomotor component (Fig. 7; Swimming (Patterns 6, 7), Sitting (Patterns 8, 9)), but all four of those patterns were also observed, at various levels of frequency, with other locomotor components. Furthermore, none of the resulting patterns contained any bands, spots, stripes, or waves on the arms.

## Discussion

In this study, we were able to observe the patterning repertoire of freely behaving, sexually mature *M. pfefferi* in their home aquarium. These observations led to the description of 7 textural, 14 postural, 7 locomotor and between 42 and 75 chromatic dorsally-visible components commonly observed in this group of animals. From these data, we found that these components combine into 11 distinct body patterns used by this population of *M. pfefferi*.

Overall, the maximum total number of chromatic, postural, textural and locomotor components identified in this population (103) is more than double the number found in the cuttlefish with the most complex patterning repertoire studied to date: *Sepia officinalis* (54 components total: Hanlon & Messenger, 1988). One of the reasons we think this might be the case is that our methods of collecting these data deviated slightly from those used to produce ethograms in other species of cephalopod. Specifically, we split apart components that may have been grouped by other ethologists. The implications of this methodological variation when describing the body patterning repertoire of this population of *M. pfefferi* are discussed below.

The most notable difference between the methods described herein and those used by other ethologists involves our definition of a chromatic component. Our separation of components by color (as seen by the human eye) and their repetitive and consistent expression has led to the description of twice as many chromatic components (75; Fig. 3, Expanded List) as any other cuttlefish species (35 in *S. officinalis* Hanlon & Messenger, 1988, 12 in *Sepia papuensis* Roper & Hochberg, 1988, 11 in *Sepiola affinis* Mauris, 1989). Our attempts to make our data comparable to previous studies (Fig. 3, Condensed List) still result in a larger list of chromatic components (42) than are found in other cuttlefish species. However, caution should be used when comparing the number of components observed in various species as there are many differences in the descriptions of chromatic components among ethograms.

Many published ethograms describe the chromatic components of a species and also list “variations” of certain components. This term appears to be used to group similar-looking expressions of coloration on the skin as one component instead of many, though, to our knowledge, no formal distinction between a “component” and a “component variation” exists. Without a consistent rule to describe whether an expressed area of coloration should be defined as an independent component or a variation of a similar component, there are many inconsistencies among studies. For example, an ethogram of *Doryteuthis plei* depicts a single component “Arm spots” with 4 variations. Each variation shows a different number and location of spots on the animal’s arms (Postuma & Gasalla, 2015: Fig. 2). However, other studies depict “Arm spots” as a single component with no variations (Hanlon et al., 1999; Hanlon & Messenger, 1988; Hanlon, Smale & Sauer, 1994) and in the original description of *M. pfefferi*, “Arm spots” were split into three separate components (Arm IV white spots, Arm III white spots, and Arm I white spots) with no variations (Roper & Hochberg, 1988: Table 6). In another example, the component we termed “Arm tips” has been described in other species as both a variation of “Arms/head” (Hanlon et al., 1999) and an independent component (Byrne et al., 2003; Dubas et al., 1986; Hanlon, Smale & Sauer, 1994; Trueblood et al., 2015). These inconsistencies make inter-observer comparisons of chromatic component counts difficult.

Using the hierarchical system of body patterning, a possible solution to the inconsistent separation of components may be to evaluate the morphological and physiological units of an animal. However, for studies in which only whole-animal observations are possible (e.g., the animal is on display or the animal is observed solely through ROV footage) this type of microscopic analysis is currently impossible. An alternative may be to assess which groupings of coloration are necessary for the successful camouflage or communication of the pattern an animal is displaying. Necessary components (or essential components) could then be grouped separately from those that are unnecessary to a pattern’s presentation. Unfortunately, few data on this topic exist. While we did not perform any experimental studies to assess whether any components were essential in this population of animals, some areas of coloration were consistently displayed by the animals in different contexts from similar-looking coloration. For example, Pattern 11 (Fig. 7) was consistently expressed with “Arm tips (dark)” (100% of observations) but was never observed with dark Arms or Head. As previously mentioned these three components have been described as variations of the component “Dark arms/head” in another ethogram and were thus combined in our Condensed List of components (Fig. 3). If these components were used interchangeably by the animal, then considering them as variations of a single component may be appropriate, but the animals’ consistent use of Arm tips (dark) leads us to believe that these should be treated as separate components for this species.

We found this same consistent expression of coloration amongst similar components that only differed in their color (as seen by the human eye). Functionally, the production of yellow coloration on a cephalopod’s skin is likely the result of chromatophore expansion whereas the production of white is caused by chromatophore retraction. However, both colors cause a strong contrast to nearby dark areas, and thus were grouped as a single “light” component in our Condensed List. Head spots, the circles of color between the eyes and arms present in 8 of our 11 patterns (Fig. 7), were expressed in both white and yellow, leading us to define them in our Expanded List as two separate components. If the color wasn’t necessary to the pattern’s presentation, one would expect to see a roughly even distribution of white and yellow Head spots displayed in each pattern. However, in this study, each pattern that involved Head spots was almost exclusively expressed in only one color. For instance, 81% of the video clips sorted into Pattern 10 involved Head spots and in 100% of those observations the Head spots were white. Alternatively, in Pattern 1, 94% of the clips involve an animal with Head spots and in 99% of those observations the Head spots were yellow. This uneven use of white and yellow Head spots is present in each of the 8 patterns that involve this component. We have no reason to believe that the visual system of *M. pfefferi* differs drastically from other color-blind cephalopods (Mäthger et al., 2006), making this consistent variation in component expression particularly perplexing.

Given these observations, it is possible that if Arm tips were considered a variation of Dark arms/head and if white and yellow components were grouped together as “light” we would miss some subtle but potentially important pieces to *M. pfefferi* body patterning. We suggest that until more data can be collected regarding *M. pfefferi* visual systems, morphological and physiological units, chromatic component use in the wild, and component essentiality that any further body pattern observations of this species be described in terms of a live animal’s consistent expression of groupings of coloration, similar to our “Expanded List.” Collecting and presenting data, including raw data, in this way allows for future updates and re-groupings of components to reflect future discoveries as they are made without losing potentially important features.

Using this suggestion, we analyzed our Expanded chromatic components in combination with the textural, postural and locomotor components observed in this study using the AutoClass@IJM system and identified 11 Patterns (Fig. 7). This number is consistent with other cephalopods which exhibit between 2 and 16 patterns (for review, see Borrelli, Gherardi & Fiorito, 2006; Hanlon & Messenger, 1996). A similar study which utilized AutoClass clustering algorithms to analyze the number of body patterns of the common cuttlefish, *S. officinalis*, found 12 distinct patterns (Crook, Baddeley & Osorio, 2002). This number is very similar to the 13 patterns identified through extensive and rigorous field and laboratory observations of the same species (Hanlon & Messenger, 1988). The AutoClass algorithm was originally developed by the Bayesian Learning Group at the NASA Ames Research Center to automatically find the “natural classes” in large datasets (Cheeseman & Stutz, 1996; Hanson, Stutz & Cheeseman, 1991). For *M. pfefferi* body patterning, we found this program to be particularly useful due to the large total number of chromatic, postural, textural and locomotor components (103). Once these classes are reported by the software, they still need to be interpreted by a human with extensive knowledge of the subject.

One such area of needed interpretation in this study lies in what was not classified as a separate pattern in *M. pfefferi*: one that would be useful for camouflage in their home aquarium. While we did not formally investigate camouflage in this study, the fact that 91% of the identified patterns are primarily dark in coloration despite the pale crushed coral substrate of their environment suggests to us that these patterns are likely to be very conspicuous, at least to the human eye. We think it is unlikely that this is an artifact of the AutoClass program, as the program did differentiate between various cryptic patterns in *S. officinalis* (Crook, Baddeley & Osorio, 2002). The only pattern that would have limited the amount of contrast between the animal and the substrate in this environment was Pattern 11, but this pattern was displayed primarily by males during mating attempts (60% of observations) or conspecific aggression (24% of observations). It is possible that the almost total lack of cryptic behaviors observed in this population is an artifact of their aquarium environment. Substrate, lighting, crowding levels, inbreeding depression and the distraction of nearby humans each could have influenced their patterning behavior in this study. The two reported studies of *M. pfefferi* in the wild note high levels of crypsis in their natural environment (Reid, Jereb & Roper, 2005; Roper & Hochberg, 1988), but any further studies of *M. pfefferi* in the wild or in a semi-natural environment are necessary to better understand their behavior.

A final noteworthy observation from this population of *M. pfefferi* in their home aquarium involves the use of “traveling” chromatic components. These include the “traveling wave” components initially described by Roper & Hochberg (1988) and studied intensively by Laan and colleagues (2014). Unlike the “passing cloud” component exhibited by *S. officinalis*, (Fig. 4.6 in Hanlon & Messenger, 1996), the traveling waves of *M. pfefferi* and their close relative *M. tullbergi* occur in 4–5 distinct locations on the mantle (See ‘Expanded List’ in the Results section). The Passing cloud of *S. officinalis* is also primarily observed during hunting and has been interpreted by human observers as “stop and watch me” when directed towards prey (p.127 Hanlon & Messenger, 1996). Alternatively, the traveling waves in *M. pfefferi* (this study) and *M. tullbergi* (Laan et al., 2014) have been observed consistently for long periods of time during a variety of activities. In fact, 8 of the described 11 body patterns exhibited by this population of *M. pfefferi* contained traveling waves in at least one of the described regions (Fig. 7; Patterns 2, 3, 4, 5, 6, 7, 9 and 10), and three patterns contained traveling waves in all four regions (Fig. 7; Patterns 3, 4, and 9). We suggest that the repeated, consistent use of these components may indicate that traveling waves do not carry the same communicative information in this population of *M. pfefferi* as traveling components in other species of cephalopod.

## Conclusion

In this study, we provide the first observations of a population of sexually-mature, freely-behaving, aquarium-raised *M. pfefferi* in their home environment. We hope that the chromatic, textural, postural and locomotor components as well as the 11 body patterns described herein provide detailed groundwork that will be helpful in future studies of the species. To make our observations as adaptable to future discoveries as possible, we have made both an editable component database and our raw binary component data (File S1 and 10.6084/m9.figshare.1509930) freely available and encourage other researchers to use these resources as needed.

## Supplemental Information

10.7717/peerj.2035/supp-1 File S1 Interactive database of *Metasepia pfefferi* components For the reader’s convenience, this file is available for download as a PDF and as an Adobe Illustrator file. Instructions to utilize these files are as follows: in the PDF: open using Adobe Acrobat Pro. Click the “Layers” button to expose the available layers. Check or uncheck each box next to each layer to see it appear on the cuttlefish. The Adobe Illustrator file is available on figshare (10.6084/m9.figshare.1509930). In the Adobe Illustrator file: open using Adobe Illustrator. Open the layers tab and toggle the visibility on each layer to reveal the various components on the cuttlefish.

10.7717/peerj.2035/supp-2 Video S1 *Metasepia pfefferi* walking with wide arms The adult *M. pfefferi* moves throughout its environment to eat prey using the locomotor component “Walking with wide arms”. Note the bend to the animal’s walking arms and the extension of the feeding tentacles. This method of locomotion was seen during feeding and non-feeding times.

10.7717/peerj.2035/supp-3 Video S2 *Metasepia pfefferi* walking with tall arms This adult *M. pfefferi* moves throughout its environment using the locomotor component “Walking with tall arms”. Note the animal’s extended walking arms. The animal also exhibits an acute cryptic body patterning for 5 s between two conspicuous chronic body patterns.

## Additional Information and Declarations

### Data Availability

The following information was supplied regarding data availability: Figshare: 10.6084/m9.figshare.1509930.
