# Supplementary figures and images for: Investigating body patterning in aquarium-raised flamboyant cuttlefish (Metasepia pfefferi)
Source: PeerJ. 2016 May 17;4:e2035. doi: 10.7717/peerj.2035 (PMC4878381; doi:10.7717/peerj.2035)

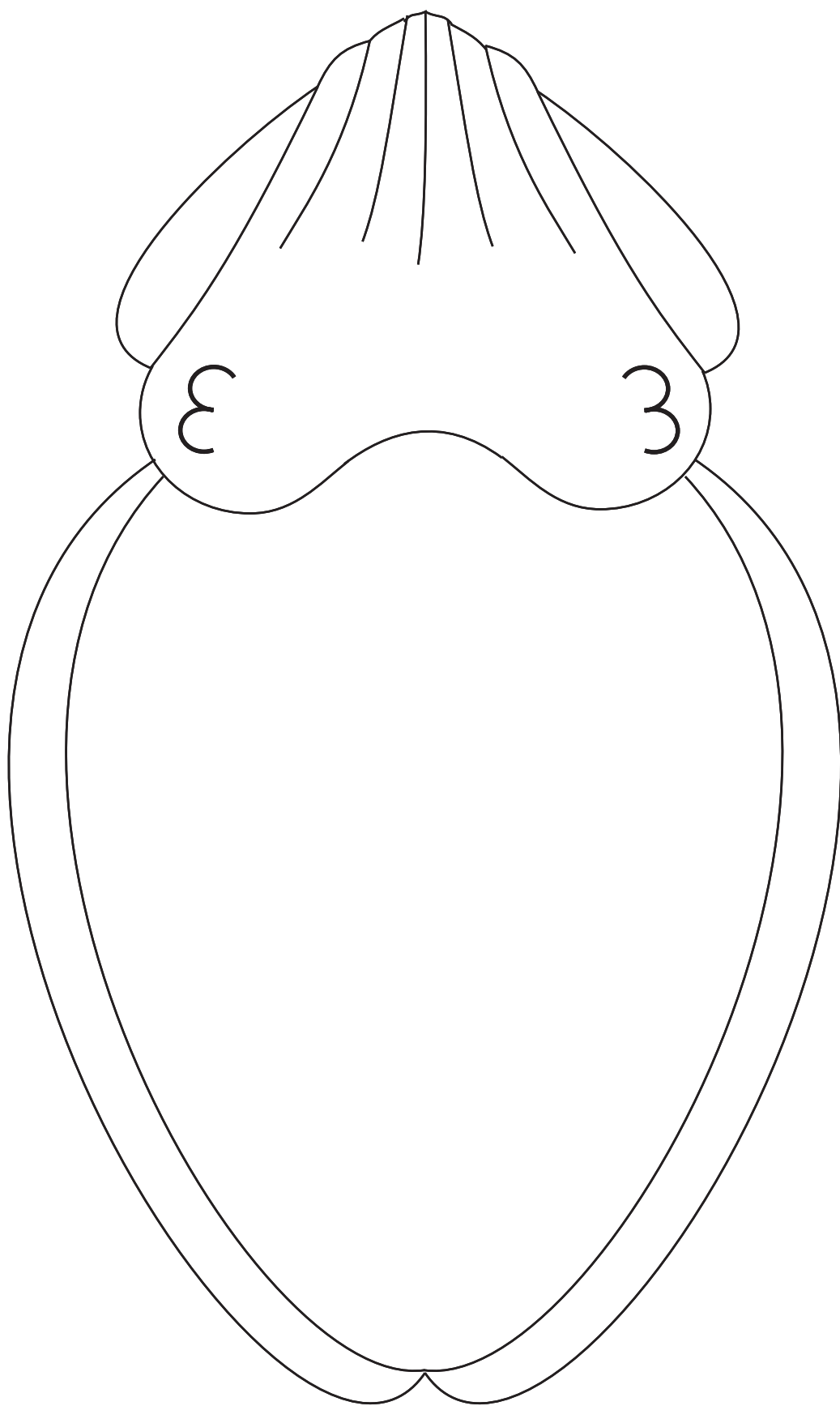

Supplement: File S1 — For the reader’s convenience, this file is available for download as a PDF and as an Adobe Illustrator file. Instructions to utilize these files are as follows: in the PDF: open using Adobe Acrobat Pro. Click the “Layers” button to expose the available layers. Check or uncheck each box next to each layer to see it appear on the cuttlefish. The Adobe Illustrator file is available on figshare (10.6084/m9.figshare.1509930). In the Adobe Illustrator file: open using Adobe Illustrator. Open the layers tab and toggle the visibility on each layer to reveal the various components on the cuttlefish. [file peerj-04-2035-s001.pdf]
